# The role of stretch, tachycardia and sodium‐calcium exchanger in induction of early cardiac remodelling

**DOI:** 10.1111/jcmm.15504

**Published:** 2020-06-22

**Authors:** Natasa Djalinac, Senka Ljubojevic‐Holzer, Ingrid Matzer, Ewald Kolesnik, Katharina Jandl, Birgit Lohberger, Peter Rainer, Akos Heinemann, Simon Sedej, Dirk von Lewinski, Egbert Bisping

**Affiliations:** ^1^ Department of Cardiology Medical University of Graz Graz Austria; ^2^ Institute of Experimental and Clinical Pharmacology Medical University of Graz Graz Austria; ^3^ Ludwig Boltzmann Institute for Lung Vascular Research Graz Austria; ^4^ Department of Orthopedics and Trauma Medical University of Graz Graz Austria

**Keywords:** Calcium/Calmodulin‐dependent protein kinase II (CaMKII), cardiac remodelling, ET coupling (ETC), hypertrophy, mechanical stretch, neonatal rat ventricular cardiomyocytes (NRVCM), RNA expression, sodium‐calcium exchanger (NCX), tachycardia

## Abstract

Stretch and tachycardia are common triggers for cardiac remodelling in various conditions, but a comparative characterization of their role in the excitation‐transcription coupling (ETC) and early regulation of gene expression and structural changes is lacking. Here, we show that stretch and tachycardia directly induced hypertrophy of neonatal rat cardiac myocytes and also of non‐myocytes. Both triggers induced similar patterns of hypertrophy but had largely distinct gene expression profiles. *ACTA1* served as good hypertrophy marker upon stretch, while *RCAN1* was found increased in response to tachycardia in a rate‐dependent fashion. Mechanistically, several calcium‐handling proteins, including the sodium‐calcium exchanger (NCX), contributed to ETC. Phosphorylation of the calcium/calmodulin‐dependent protein kinase II (CaMKII) was elevated and occurred downstream of NCX activation upon tachycardia, but not stretch. Microarray profiling revealed that stretch and tachycardia regulated around 33% and 20% genes in a NCX‐dependent manner, respectively. In conclusion, our data show that hypertrophy induction by stretch and tachycardia is associated with different gene expression profiles with a significant contribution of the NCX.

## 1 INTRODUCTION

Cardiac remodelling describes the gradual functional and structural changes in which the heart undergoes under pathological conditions.^1^ Excitation‐transcription coupling (ETC) is a very early key feature of this remodelling process and consists of the activation of ion‐handling molecules at the sarcolemma followed by altered fluxes of Na^+^ and Ca^2+^, which trigger downstream signalling cascades that eventually target transcription with subsequent structural changes.^2^, ^3^ Hypertrophy is one of the earliest structural features to be recognized and is an important prognostic marker.^4^ Several calcium‐ and sodium‐handling molecules have been found to be involved in ETC and hypertrophy.^5^, ^6^ This is why we addressed the following in our report: sodium‐calcium exchanger (NCX), sodium‐proton exchanger (NHE), sodium channel (Nav 1.5), sodium potassium pump (Na^+^/K^+^ ATPase), L‐type calcium channel (LTCC), transient receptor potential channel (TRP), calcium/calmodulin‐dependent protein kinase II (CaMKII) and calcineurin.

The aim of our study was to characterize in detail the similarities and the differences of two clinically important remodelling triggers: stretch and tachycardia. In primary rat cell culture (of myocytes and non‐myocytes), we compared their modification of the cellular phenotype, their underlying ET coupling and genome‐wide RNA expression profile.

Stretch is the cellular equivalent to cardiac pressure overload which is present in various clinical conditions such as hypertensive heart disease.^7^ In rat cell culture, cyclic stretch on flex membranes has been described as a relevant in vitro model to mimic pressure overload^8^

Tachycardia is also a potent trigger of cardiac remodelling.^9^, ^10^ If it is sustained over long term, it can result in a heart failure phenotype described as tachycardiomyopathy.^11^ The phenotype might be similar, but its better reversibility and its typically normal wall thickness distinguish it from pressure overload. Hypertrophy, if present at all, was considered as a potential secondary response to increased wall stress after pacing‐induced cell death in remote parts of the heart.^12^

## 2 MATERIAL AND METHODS

Detailed description of the experimental procedures is found in the Extended Material and Methods section of the Supplemental Material.

### 2.1 Cell culture

The isolation of primary neonatal rat ventricular cardiomyocytes (MC) and cardiac non‐myocytes (NMC) was conducted on 1‐ to 3‐day‐old neonatal Wistar rats. The procedure was approved by the institutional local animal care and use committee.

### 2.2 Mechanical strain (stretch)

The Flexcell FX5K Tension System (Flexcell International Corp, Burlington, USA) was used to apply a mechanical cyclic tensile strain of 120% at 0.5 Hz frequency on MC and NMC cells for up to 72 hours. Cells grown under the same conditions but in the absence of the strain protocol served as control.

### 2.3 Electrical field stimulation (Tachycardia)

Field stimulation was applied using a C‐Pace EP culture pacer (IonOptix, Westwood, USA) at the stimulation rates 1‐8 Hz, 5‐8 V/cm electrode distance and 4 ms pulse duration for 3‐48 hours as indicated. Stimulation voltage was defined as a 2‐fold stimulation threshold at which at least 50% of cells responded with contraction. Unstimulated cells (0 Hz) and normal rate pacing cells (1 Hz) served as controls.

### 2.4 Antagonist treatment

Cells were incubated with or without selective inhibitors (for details, please refer to the Extended material and methods) for 1 h before being subjected to stretch or tachycardia.

### 2.5 Quantitative real‐time PCR (qPCR)

All qPCR experiments were performed with a LightCycler® 480 Instrument (Roche Applied Sciences, Penzberg, Germany) following the MIQE guidelines. Primer and probe sequences are provided in the Table S1. Efficiency correction for all primer sets was performed using GenEx 5.4.4. (MultiD, Gothenburg, Sweden). Relative gene expression was determined using the 2^‐ΔΔCt^ quantification method.

### 2.6 Microarray hybridization

Microarray hybridization was carried out using Rat Gene 2.0 ST Array (Thermo Fisher Scientific, Wilmington, USA). Data pre‐processing and filtering were done with the Partek^®^ Genomics Suite^®^ software (V.6.6). Validation was performed by qPCR for selected genes. Bioinformatical assessment was done with Ingenuity^®^ Pathway Analysis software (Qiagen, Hilden, Germany). The microarray data can be accessed via the GEO database under the series number GSE135172.

### 2.7 Immunofluorescent staining

After the stimulation period, cells were fixed in 4% PFA, stained for specific antigens using primary antibodies against desmin (DAKO), DDR2 (Santa Cruz), NCX (SWANT), P4HB, Vimentin, P‐CaMKII, ADAMTS13 (Abcam), incubated with fluorophore‐conjugated secondary antibodies and visualized on Zeiss LSM 510 Meta and Nikon A1 confocal microscopes. The images were analyzed using ImageJ software (ImageJ 1.46r, NIH, USA).

### 2.8 Flow cytometric staining

NCM were stained with eBioscience^™^ Fixable Viability Dye eFluor^™^ 506 dye to assess viability and incubated using NCM specific antibodies, namely APC/Fire 750 anti‐rat CD45 antibody (BioLegend) and PE anti‐rat CD31 antibody (Miltenyi Biotec). Fluorescent signal was acquired with a Cytoflex S (Beckman Coulter, USA).

### 2.9 Statistical analysis

Statistical analysis was performed using GraphPad Prism 8 (GraphPad Software, Inc, La Jolla, USA). Results are represented as mean ± SEM *P*‐value < 0.05 was considered statistically significant.

## 3 RESULTS

### 3.1 Effects of stretch and tachycardia on morphology in cardiac myocytes and non‐myocytes

First, we investigated the morphology and size of cardiac myocytes (MC) and non‐myocytes (NMC) in response to stretch (ST) and tachycardia (TC). On the one hand, immunofluorescent analysis of the CM culture revealed 70% desmin‐positive cells, confirming a myocyte‐enriched culture. The vast majority of CM maintained frequency‐dependent excitation‐contraction coupling throughout the interventions as demonstrated upon regular exchange of the cell culture media. On the other hand, the NCM culture consisted of 95% cells positive for the NCM selective marker P4HB, while negative for the cardiomyocyte‐specific marker desmin. We further explored the cell type composition of NMC by flow cytometry and immunochemistry (Figure S1), which revealed that the majority of NMC were fibroblasts (90%), and only small portions were formed by endothelial cells (5%), inflammatory cells (4%) and others (1%).

**FIGURE 1:**
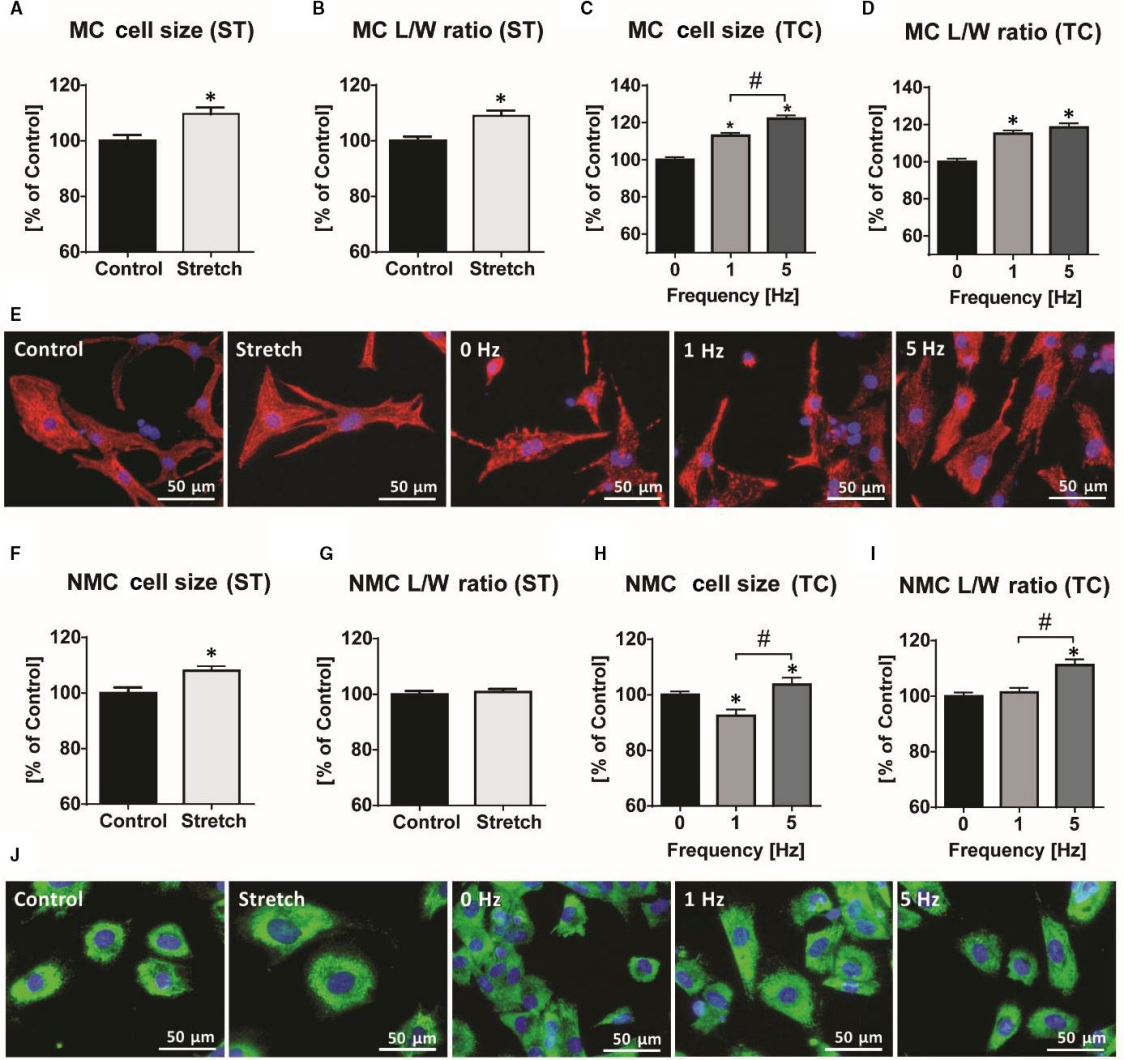
Morphological characteristics of neonatal rat ventricular myocytes (MC) and non‐myocytes (NMC) upon mechanical stretch (ST) and tachycardia (TC). A, Induction of MC hypertrophy and B, elongation presented as length‐to‐width ratio after 72 h of ST. C and D, Frequency‐dependent changes in MC size and elongation after 48 h of tachycardia. F and G, ST induces NMC hypertrophy after 72 h without effects on cell elongation (length‐to‐width ratio). H, I, Increase in NMC cell size and elongation after TC (5 Hz). E, J, Immunofluorescent staining of representative fields from bio‐flex and 35 mm dishes. ^*^ *P *< .05 vs control ^#^ *P *< .05 for pairwise comparison. Average cell count: n = 700‐1000 per group

Stretch moderately increased MC surface area for 10%, reaching significance the earliest by 72 hours (Figure 1A, *P* < .001, n = 1800). This was accompanied by cell elongation as evidenced by 8% increase of the cell length‐to‐width ratio (Figure 1B, *P* < .001). Cell adhesion and viability appeared unchanged at this time‐point (Figure 1E).

Tachycardia induced a complex response in MC, which unexpectedly increased in size after 48 hours at 1 Hz (ie a non‐tachycardic stimulation). This was associated with a pronounced eccentric pattern compared to unstimulated controls. Stimulation at 5 Hz further increased the cell size, indicating a frequency‐dependent hypertrophy with an identical eccentric pattern (Figure 1C‐D). Cell viability gradually decreased under tachycardia, which prevented us to generate reproducible data at 8 Hz stimulation after 48 hours (data not shown).

### 3.2 Cell type dependence of Stretch‐ and Tachycardia‐induced gene regulation

The enriched MC and pure NMC were used to address cell type dependence of trigger induced gene expression changes. We picked 5 marker genes which all have been linked to hypertrophy in a variety of in vivo and in vitro models. The selection is discussed in detail in paragraph 4.2.

**FIGURE 2:**
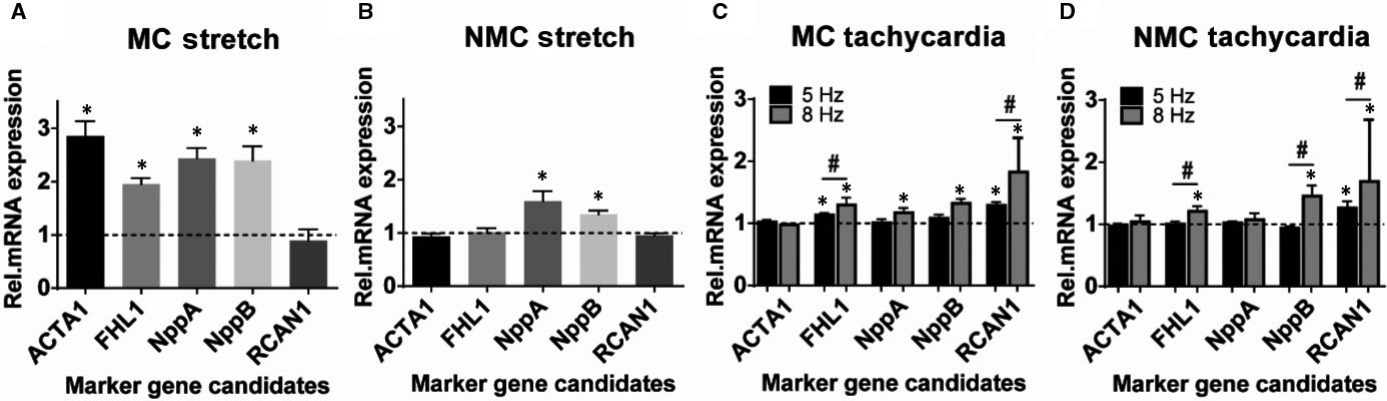
Gene expression changes induced by stretch (ST) and tachycardia (TC) in neonatal rat ventricular myocytes (MC) and non‐myocytes (NMC). A, Up‐regulation of remodelling marker genes after 24 h of ST in MC. B, Up‐regulation after 24 h of ST in NMC. C, D, Rate‐dependent gene expression changes after 3 h of TC in MC (C) and NMC (D). Dashed line represents the baseline expression of the corresponding control. ^*^ *P *< .05 vs control, ^#^ *P* < .05 for pairwise comparison. n = 4‐13 cell cultures

Stretch in MC resulted in a time‐dependent up‐regulation of marker genes with a peak effect at 24 hours (Figure S2). At the 24‐hours time‐point, 4 out of 5 marker genes were up‐regulated (Figure 2A) with the gene *ACTA1* showing the most pronounced response. On the contrary, non‐myocytes (NMC) showed only a slight up‐regulation of the natriuretic peptides without expression changes of any other investigated marker gene at 24 hours (Figure 2B).

Tachycardia in MC resulted in largely unchanged gene expression levels when performing a timeline screening (Figure S3). However, *RCAN1* showed a slight but significant up‐regulation at 3 hours. Therefore, our further assessment was performed exclusively at this single time‐point but with two distinct stimulations rates, 5 Hz and 8 Hz. Under these settings, cell viability was only slightly decreased at maximum tachycardic rate versus 1 Hz (61.5% vs 66.5%, *P* < .05). We found a clearly rate‐dependent up‐regulation: *FHL1* and *RCAN1* were mildly up‐regulated at 5 Hz and significantly stronger up‐regulated at 8 Hz. The natriuretic peptides *NppA* and *NppB* were also mildly up‐regulated but reached level of significance only with 8 Hz stimulation (Figure 2C). In NMC, effects were less pronounced than in MC but still clearly present: 5 Hz stimulation induced up‐regulation of *RCAN1*, and 8 Hz stimulation resulted in up‐regulation of *FHL1, NppB* and *RCAN1.* The effect for *RCAN1* was rate‐dependent (Figure 2D).

### 3.3 Excitation‐transcription coupling of Stretch‐ and Tachycardia‐induced gene regulation

**FIGURE 3:**
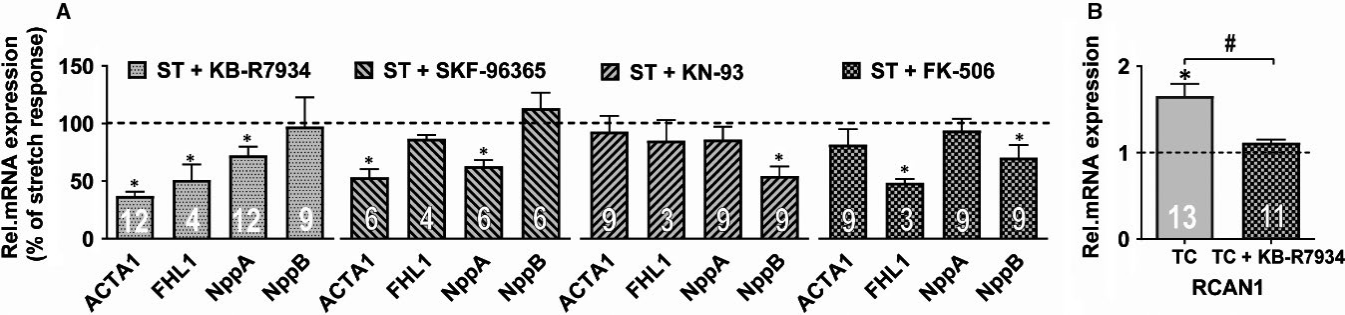
Excitation‐transcription (ET) coupling in stretch (ST) and tachycardia (TC). The experiments were performed in 4 groups: stretch/tachycardia + vehicle, control + vehicle, stretch/tachycardia + inhibitor and control + inhibitor. The treatments were normalized to their corresponding control group (group 1 to group 2; group 3 to group 4). A, Marker gene expression upon inhibition of sarcolemmal and intracellular signalling targets in stretched myocytes. B, *RCAN1* gene expression in the absence and presence of the NCX inhibitor KB‐R7934 after 3 h of TC stimulation. The dashed line represents the baseline expression of the corresponding controls. ^*^ *P* < .05 ^#^ *P* < .05 (stretch + inhibitor vs control + inhibitor). Numbers in bars represent the number of technical replicates

To gain mechanistical insights for upstream regulators of our selected marker genes, we performed inhibition of 7 sarcolemmal and 2 intracellular Na^+^‐ and Ca^2+^‐handling proteins. We found that out of the sarcolemmal protein inhibitors KB‐R7934, a reverse mode inhibitor of the sodium‐calcium exchanger (NCX) significantly blocked the up‐regulation of the strongest up‐regulated gene *ACTA1* (*P* < .001, Figure 3A). In addition, it significantly attenuated the up‐regulation of *FHL1* and of *NppA*. SKF‐96365, a broad range TRP channel inhibitor, significantly repressed *ACTA1* and *NppA* up‐regulation (*P* < .05). All other sarcolemmal protein inhibitors only showed non‐significant trends in our screening approach (Figure S4).

The calcium sensors CaMKII and calcineurin were evaluated as the intracellular downstream candidate molecules for ET coupling: We found for CaMKII that its low‐specificity inhibitor KN93 blocked the stretch‐mediated up‐regulation of *NppB* (*P* < .05). Also, the calcineurin inhibitor FK506 repressed *FHL1* and *NppB* up‐regulation (Figure 3A).

We tested whether inhibition of the NCX, as the most relevant target in stretch, had also effects in tachycardia‐induced gene regulation: We found that the up‐regulation of *RCAN1* after 3 hours of tachycardia stimulation (Figure 3B) was significantly reversed to near‐baseline expression (*P* < .001).

### 3.4 Increased NCX expression by Stretch, increased CaMKII phosphorylation by Tachycardia

**FIGURE 4:**
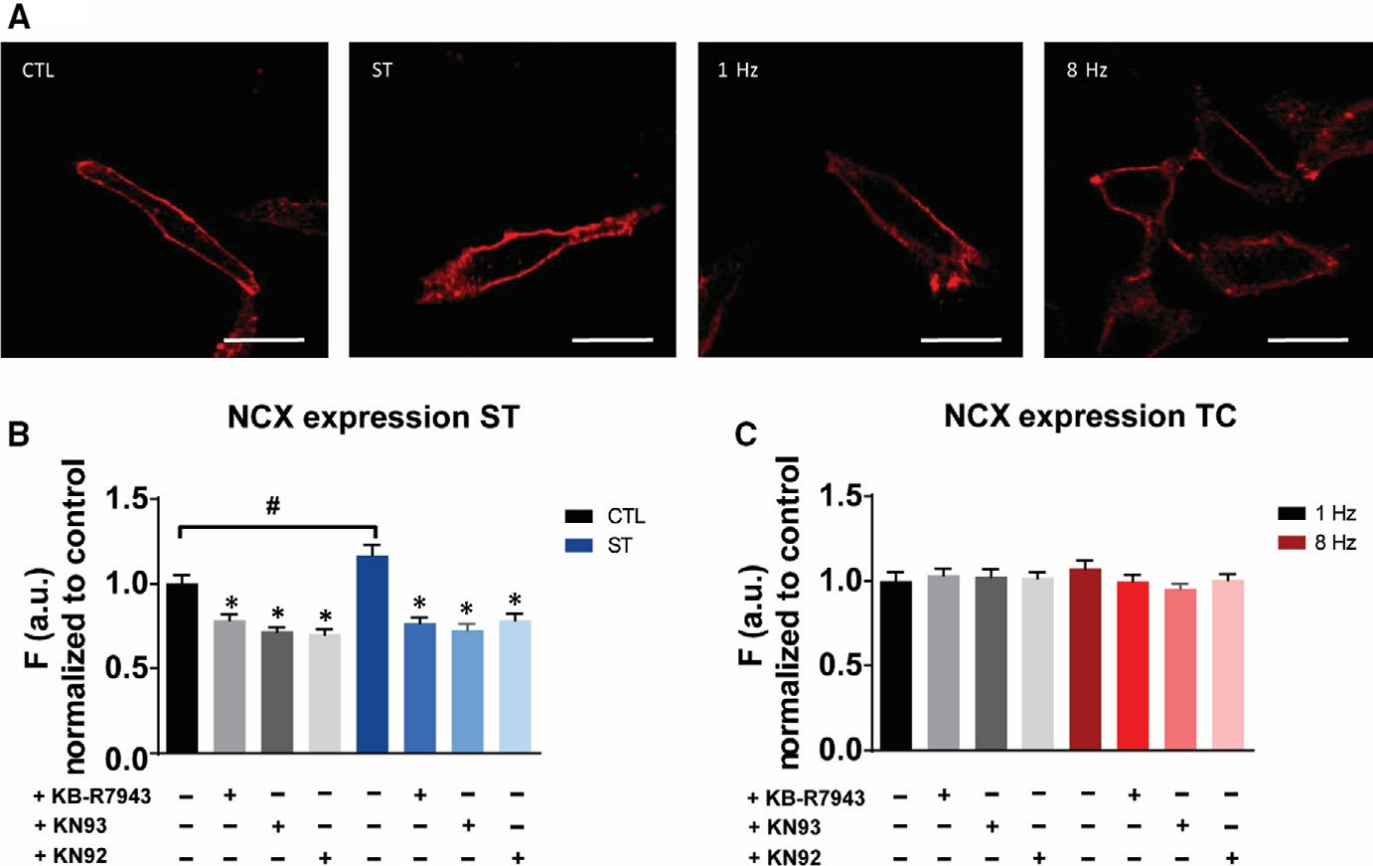
Differential NCX protein expression between stretch (ST) and tachycardia (TC). A, Immunofluorescent staining of representative fields from bio‐flex and 35 mm dishes, scale bar = 20 µm. B, Increased NCX expression after 24 h of stretch is independent of the CaMKII signalling pathway. C, Tachycardia (8 Hz, 3 h) does not alter NCX expression. ^*^ *P* < .05 vs control/ vs ST, ^#^ *P *< .05. Average cell count n = 500‐600 per group

Having shown that NCX activity is involved in ET coupling for stretch and tachycardia, we were interested whether also its protein expression levels might have changed. NCX expression levels were measured by immunofluorescence (Figure 4A) and found to be increased upon 24 hours of stretch (Figure 4B). This effect was abolished by application of the NCX inhibitor KB‐R7943 suggesting that increased NCX expression is a consequence of increased activity. Furthermore, NCX up‐regulation was blocked by the CaMKII inhibitor KN93, but also by its inactive analogue KN92, why we consider this as an evidence of an off‐target effect of the KN inhibitors. In contrast to stretch, tachycardia for 3 hours did not induce any expression change (Figure 4C).

**FIGURE 5:**
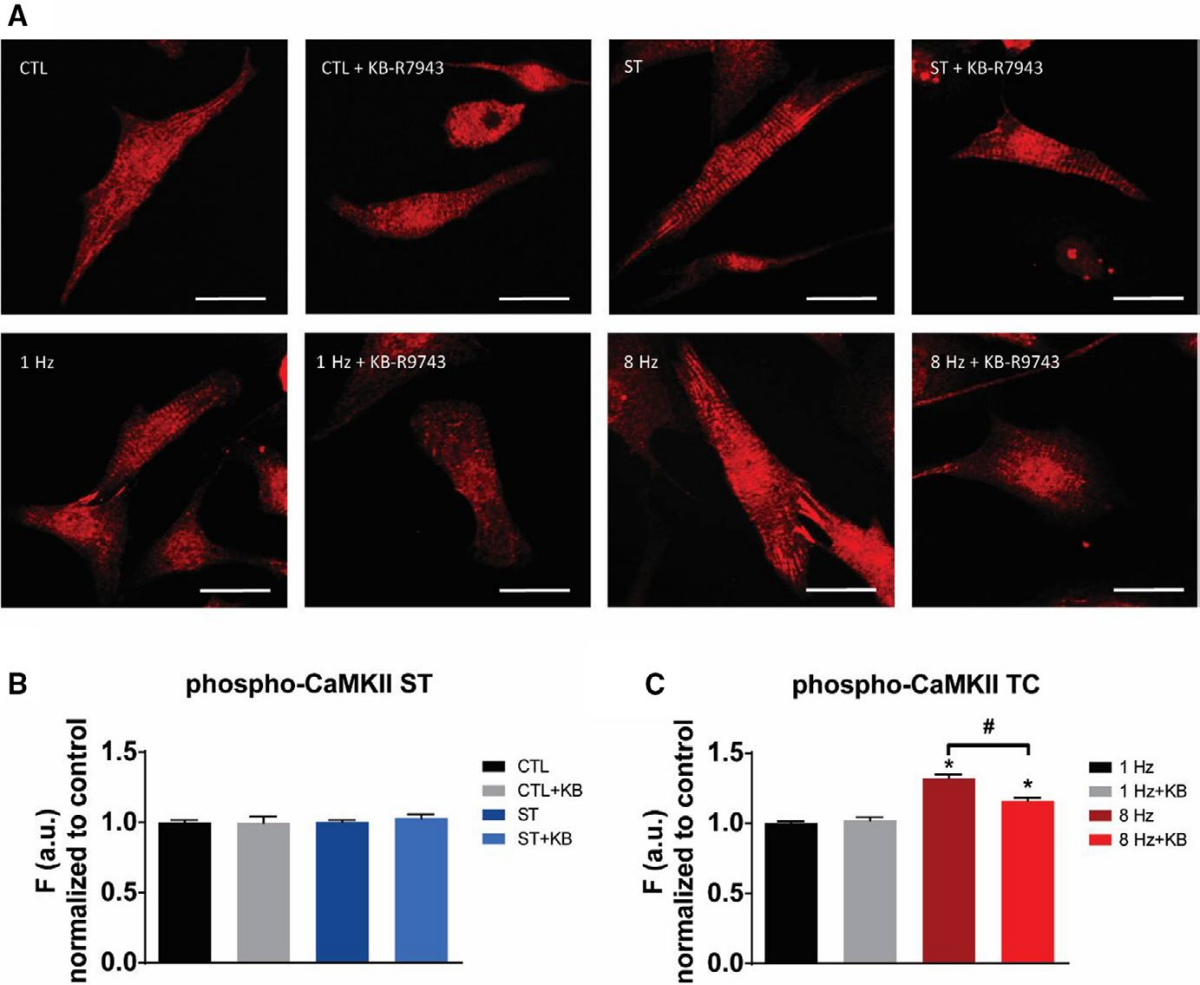
Effect of stretch (ST) and tachycardia (TC) on CaMKII phosphorylation at T286. A, Immunofluorescent staining of representative fields from bio‐flex and 35 mm dishes, scale bar = 20 µm. B, Unchanged phosphorylation of CaMKII after 24 h of ST. C, Increased phosphorylation of CaMKII upon 3 h of tachycardia (8 Hz). *P < .05 vs control, ^#^ *P* < .05. Average cell count n = 1000‐1500 per group

With the problem of off‐target KN‐inhibitor effects in mind, we now went to investigate CaMKII activity more directly by measuring phosphorylation at its T286 autophosphorylation site (Figure 5A). There was no sign of CaMKII activation during stretch (Figure 5B) which confirmed that the previously mentioned effects of the KN93/92 inhibitors were off‐target. However, with tachycardia (8 Hz) we observed an increase in phosphorylation of 32% with significant (partial) inhibition by KB‐R7943 (Figure 5C). This points to involvement of CaMKII downstream of NCX in tachycardia but not stretch.

For a potential negative upstream regulator of CaMKII in tachycardia, we checked expression levels of ADAMTS13 with respect to a previous report with connection to arrhythmias.^13^ However, we found no changes in expression levels of ADAMTS13 in ST and TC (Figure S5).

### 3.5 Genome‐wide contribution of NCX in Stretch‐ and Tachycardia‐induced gene regulation

Microarray hybridizations were performed in myocytes (MC). Original data files are uploaded to the GEO repository under the series record GSE135172 and will be available for public access upon acceptance of the manuscript.

**FIGURE 6:**
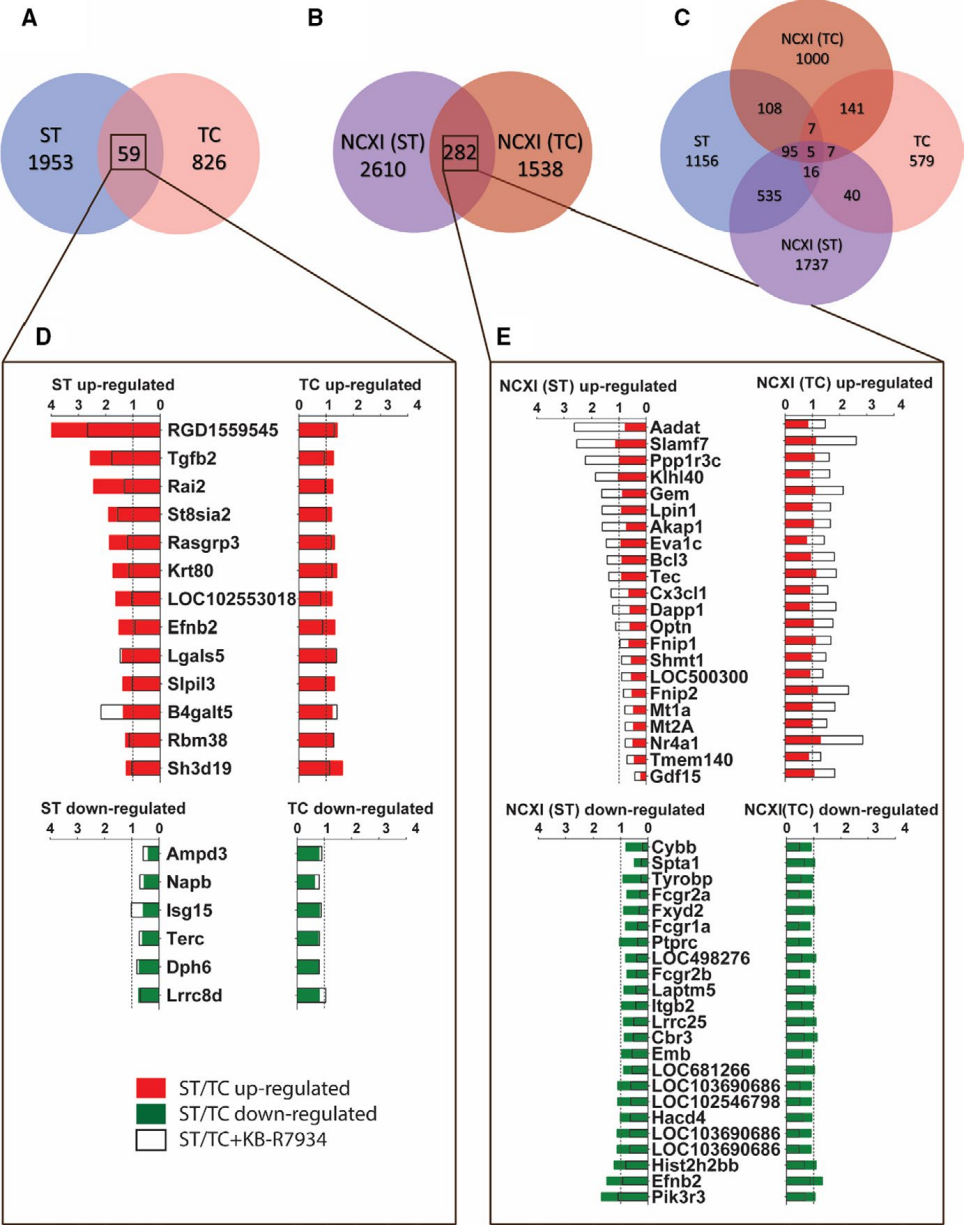
Microarray gene expression in neonatal rat ventricular myocytes (MC). The hybridization was performed in 6 groups: stretch (ST), control for ST, tachycardia (TC), control for TC, ST combined with NCX inhibition (NCXI) and TC combined with NCXI. A and D, Genes regulated either via stretch (ST) or tachycardia (TC) stimulation or by both triggers. B and E, Genes regulated by the NCX inhibitor KB‐R7934 (NCXI) in combination with ST or TC. C, Venn diagrams that display group interactions were generated from the microarray dataset thereby considering *P* < .05 and fold change cut‐off from at least 1.2‐fold up‐ or −1.2 down‐regulated (0.83 2^‐ΔΔCT^ ratio). TC/ST + KB‐R7934 (NCXI) was presented normalized to control, B, statistical significance confirmed comparing ST/TC vs ST/TC + KB‐R7934 (NCXI). n = 3 biological replicates per group

We found that ST and TC had two largely distinct gene expression profiles and a small commonly regulated fraction consisting of 59 genes (Figure 6A). Figure 6D shows genes with the highest fold changes that belong to this commonly regulated intersection. It becomes apparent that ST resulted in higher fold changes and that TC had rather marginal effects. In addition, the graph shows the effect of inhibition of the sodium‐calcium exchanger (NCXI), which inhibited the trigger‐dependent gene regulation in 47% (29 out of 59) of the common regulated genes. Among the latter were *TGFB2, RAI2, EFNB2* and *SLPIL3*, while *ISG15* was among the strongest down‐regulated genes with NCX dependency.

When NCX inhibition (NCXI) was added to the main effect of ST or TC and compared to the groups with ST or TC alone, we found a very large portion of genes regulated by NCXI (Figure 6B). Within these, a number of 282 genes were consistently regulated by NCXI regardless of the main trigger, which suggests that this intersection represents the most robust NCX‐regulated targets. The genes with the highest fold changes of this intersection are presented in Figure 6E.

The Venn diagram in Figure 6C shows the intersection of all 4 groups. It becomes evident that out of the total number of ST‐regulated genes, 651 (32%) are regulated via NCX. In the case of TC, this portion is smaller, but clearly apparent, with 160 genes (18% of total number of TC‐regulated genes). The intersection of regulation by both main triggers and NCXI are the five following genes: *SLPIL3*, *EFNB2*, *KIF14*, *CCNA2* and the sequence *TL0ACA36YA15*. A complete list of genes with a significant p‐value and a fold change cut‐off of at least 1.2 or −1.2 for either of the conditions sorted by gene symbol are provided in the Supplemental Table S2.

In order to find NCXI‐mediated expression changes, that are associated with known canonical signalling pathways, we also performed an ingenuity pathway analysis (IPA). The most relevant findings include ST‐activated cell cycle re‐entry, proliferation and motility all of which were reversed by inhibiting NCX. In terms of functional alterations, IPA revealed that ST induces cell death both via necrosis and apoptosis and impacts cellular assembly through microtubule, cytoskeleton and cytoplasm reorganization: All of the mentioned changes were reversed via NCX inhibition. A complete list of the IPA analysis is provided in the Supplemental Table S3.

## 4 DISCUSSION

Following our aim to characterize tachycardia‐ (TC) *versus* stretch (ST)‐induced early remodelling, we demonstrated that stretch and tachycardia induce hypertrophy in cardiac myocytes and non‐myocytes. ST and TC induced mainly distinct gene expression patterns with *ACTA1* as a consistent marker for ST and *RCAN1* for TC and different profiling on microarrays. Marker gene specificity to myocytes differed between both triggers. Mechanistically, we found several calcium‐handling proteins, including the NCX to be involved in ET coupling. The calcium sensor CaMKII was activated downstream of NCX in TC, while not in response to ST. Testing the contribution of NCX on genome‐wide level revealed that around 33% of the ST‐regulated genes were NCX‐dependent and 18% of the TC‐regulated ones.

### 4.1 Hypertrophy is a common hallmark of structural remodelling in all cardiac cell types upon Stretch and Tachycardia

Stretch was shown to induce hypertrophy by increased protein synthesis or altered marker gene expression^14^, ^15^ and cell size in ventricular^16^ and atrial myocytes.^17^ Our data extend previous findings by demonstrating that hypertrophy induced by cyclic stretch is not limited to cardiac myocytes, but comparably affects also non‐myocytes (NMC). By using the term NMC, we take into account all present cell populations and acknowledge that the majority consisted of the fibroblast group. Our findings are in agreement with the study by Ruwhof *et al*, who found an increase in protein synthesis as a surrogate of hypertrophy in non‐myocytes after short‐term cyclic stretch.^18^

We show, for the first time, that tachycardia‐induced hypertrophy is an early remodelling feature, which is not secondary to potential hemodynamic effects. We defined a stimulation rate of 1 Hz as control, since this was near to the average spontaneous unsynchronized beating rate of myocytes that we observed in our setting (data not shown) similar to that reported previously.^19^ Interestingly, already 1 Hz stimulation had strong effects when compared to unstimulated cells, which can be considered as prevention of atrophy rather than induction of hypertrophy^20^ as unstimulated cells are known to decline in cell size. Given the proximity of 1 Hz to the spontaneous beating rate, this effect is pacing‐dependent but not rate‐dependent. In contrast, 5 Hz showed clear rate‐dependent effects. Of note, we tested 8 Hz, a stimulation rate that is in a higher tachycardic range under both in vitro and in vivo conditions.^21^ Although stimulation up to 8 Hz was suitable for the evaluation of gene expression (Figure 2), we experienced increased cell death at the required time duration to induce hypertrophy preventing us from getting reliable cell size data for 8 Hz. This is in line with a previous report demonstrating that stimulation at this rate decreases cell survival by enhanced apoptosis in adult rat cardiac myocytes.^22^

We confirmed that tachycardia also induced hypertrophy of cardiac non‐myocytes. Previous study showed that paracrine mechanisms were directed from myocytes to fibroblasts as signal for the induction of non‐myocyte hypertrophy, for instance in canine fibroblasts.^23^ The purity of our cell culture precluded that there were any relevant myocyte‐dependent paracrine effects on non‐myocytes, suggesting that hypertrophy of cardiac non‐myocytes is a cell‐autonomous mechanism. Although fibroblasts are non‐excitable cells, they express different types of ion‐handling proteins such as K_ir_, TRPC3, sodium channels and NCX. These are sensitive to electrical stimulation and can trigger a fibroblast remodelling response in models of atrial fibrillation.^24^, ^25^, ^26^, ^27^ The potential downstream mechanisms of this remodelling response involve store‐operated calcium release, calcineurin, NFAT and miR‐26a.^25^, ^28^, ^29^

Our results from NMC upon stretch and tachycardia support the idea that both triggers target all cardiac cells instantly and independent from each other. This is particularly important at the organ level to allow for a concerted growth with sufficient NMC scaffold function and cell‐cell contacts. Paracrine mechanisms between MC and NMC might augment this reaction but are obviously not required for the remodelling response in NMC per se.

### 4.2 Marker gene expression: Trigger‐specific, myocyte preferential, rate‐dependent

We tested a selection of remodelling marker genes, which have been associated with hypertrophy in a variety of in vivo and in vitro models as well as in human disease. With this set of genes, we addressed an immediate early signalling response as also structural and functional changes that reflect an already manifesting remodelling:

Skeletal alpha (α)‐actin or *ACTA1* is involved in regulation of muscle contraction^30^ as well as cytoskeleton organization.^31^ It has been characterized as a strong marker of pathological hypertrophy^32^ with up‐regulation during the hypertrophic acute response.^33^ Four and a half LIM domain 1 (FHL1) is an interactor with the sarcomere and involved in a stress sensing system in pressure overload,^34^ thereby strongly up‐regulated in its expression.^8^, ^35^ Not only is it a marker gene but also an essential mediator of hypertrophy.^36^ The natriuretic peptides *NppA and NppB* are part of the foetal gene programme,^37^ and their up‐regulation in response to a stress trigger was documented as early as 2‐4 hours but also maintained in chronic disease models with remodelling and hypertrophy.^38^ Their clinical relevance is well established. Regulator of calcineurin 1 *(RCAN1)* is an inhibitory regulator of calcineurin and is activated early in hypertrophy.^39^, ^40^

Within this set of genes, our data revealed most pronounced responses for *ACTA1* and *RCAN1* expression under stretch and tachycardia, respectively. Both genes have been previously shown to be associated with calcineurin activity among cells of different origin.^41^ The selected time‐points, that is 24 hours for stretch and 3 hours for tachycardia, fulfilled the criteria to be as early, but also as near to the peak effect, as possible. Such an early time‐point for tachycardia as a marker of remodelling is supported by similar findings, which used rapid short‐term atrial pacing of isolated rat hearts at 4 hours^42^ and also by published time course measurements.^20^, ^43^ Our data at this time‐point also confirmed a clear rate dependency with significantly stronger effects at 8 Hz versus 5 Hz and 1 Hz.

Stretch‐induced gene expression changes in non‐myocytes affected only the natriuretic peptides, while under tachycardia the gene expression pattern was similar but less pronounced than in MC. Thus, the investigated marker genes displayed a higher degree of myocyte specificity upon stretch than tachycardia. Also, the effects in tachycardia were more transient with fading in between of 24 hours in both MC and NMC. However, this does not exclude a significant biological meaning of these gene expression changes, since they might have already induced long‐term effects on protein level at their peak effect.

Previously, it was established that protein levels of ANP and BNP can be detected only in coculture, whereas pure myocytes lack increase in their production.^44^ Our data on the ANP and BNP coding genes *NppA* and *NppB* are in agreement with this finding as our myocyte cell culture was enriched to a level that still allowed paracrine stimulation by non‐myocytes to myocytes. The proportions of up‐regulation between enriched myocytes and pure non‐myocytes led us to conclude that the majority of natriuretic peptide gene regulation derives from myocytes and not non‐myocytes.

### 4.3 ET coupling and NCX involvement in Stretch and Tachycardia

Among several inhibitory compounds linked to sarcolemmal calcium and sodium handling and intracellular signalling mediators that were tested, the Na^+^/Ca^2+^ exchanger (NCX), TRP channels, calcineurin and the protein target of KN93 showed significant involvement in stretch gene regulation. All the mediators are functionally related in a network contributing to ET coupling.

NCX regulates sodium and calcium handling simultaneously and, thus, fundamentally contributes to ET coupling.^45^ Furthermore, NCX is functionally coupled to TRPC3 and both proteins can interact to generate an arrhythmogenic stimuli.^46^ Literature findings allow to draw a potential activation cascade downstream of NCX: The proximal part of this cascade consists of activation of calcineurin^47^ and CaMKII,^48^ which have been also shown involved in tachycardia.^49^ In the light of the presented facts, prevailing effects of NCXI on gene expression and specific inhibition of the pathological reverse activity mode, we targeted NCX for further evaluation in our study. We used the NCX inhibitor KB‐R7934 at the concentration (range), which was reported to selectively inhibit the reverse mode.^50^ This mode is activated predominantly during pathological conditions promoting heart failure,^51^ and its inhibition was shown to exert beneficial effects in heart failure with preserved ejection fraction (HFpEF).^52^

### 4.4 NCX expression and CaMKII phosphorylation differ between both triggers

The stretch‐induced increase in NCX expression that was hampered by KB‐R7943 demonstrates that reverse mode NCX truly has relevance in stretch‐mediated cardiac remodelling. A positive relationship between NCX activity and expression is also well established in systolic^53^ and arrhytmogenic^54^ heart failure. In contrast, tachycardia of 3 hours did not change NCX expression, which does not exclude that it might do so at a later time‐point. But we can conclude that at 3 hours, tachycardia effects NCX activity rather by post‐translational modification.

With the CaMKII inhibitor KN93 and its inactive analogue KN92, our testing revealed an off‐target effect of these inhibitors. Indeed, both had a similar inhibitory effect on NCX expression. When we then measured CaMKII phosphorylation directly, we managed to confirm that CaMKII is not involved in stretch. Our observation coincides with a previous report of unspecific inhibition of IKr channels by KN93, which were also inhibited by KN92,^55^ suggesting that IKr might be a potential off‐target.

In tachycardia, however, our data provide evidence for a clear activation of CaMKII as indicated by its increased autophosphorylation. Based on the partial blockage of this effect by KB‐R7934, it is very likely that during tachycardia, the activation of CaMKII is in part downstream of NCX. In summary, our data reveal that this finding distinguishes stretch from tachycardia.

ADAMTS13 is a protease that regulates CaMKII phosphorylation, thereby increasing propensity towards arrhythmogenesis,^13^ and, therefore, is a very attractive candidate in a setting of acute tachycardic triggering. Although the expression levels of ADAMTS13 remained unchanged, it is possible that a post‐translational modification of the ADAMTS13 protein could promote CaMKII phosphorylation under tachycardia, which warrants further investigation.

### 4.5 The genome‐wide regulatory role of NCX in Stretch and Tachycardia

Having determined that NCX plays a major role for remodelling, we investigated the downstream gene targets of NCX at the mRNA level using microarray technology. We conclude that stretch and tachycardia mainly manifest a trigger‐specific programme with a significant distribution of NCX‐regulated genes. Substantiated by our mechanistic findings, we speculate that the expression within the tachycardia‐specific gene programme is to a certain extent CaMKII‐dependent downstream of NCX. In contrast, in stretch, involvement of NCX affects more genes and occurs independent of CaMKII. This might in part explain why many NCX‐dependent genes in response to stretch or tachycardia differed from the other.

However, there is also a fraction of commonly regulated genes by both triggers. Some individual genes of this fraction could serve as valuable biomarkers: *Efnb2* was found to be highly expressed after myocardial infarction *in vivo*.^56^ More importantly, *Efnb2* up‐regulation was successfully reversed following NCX inhibition in vitro. *Ccna2* is involved in the cell cycle re‐entry and contributing to hyperplasia,^57^ while *Kif14* ^58^ is also involved in the cell cycle regulation promoting cell proliferation. The expression directions of both genes correlate with the predictions of ingenuity pathway analysis (IPA). Interestingly, genes in relation to cardiovascular disease such as *TGFß2, Rasgrpr3 and Isg15* are subjected to significant regulation in response to both stretch and tachycardia and show a trend towards NCX dependency.

Our bioinformatical predictions by IPA are supported by previous findings, which show that stress, such as pressure overload *in vivo* ^59^ or cyclic stretch *in vitro*,^60^ enables cardiomyocyte proliferation as a compensating mechanism. Proliferation is directly associated with cell cycle alterations, while cell motility is mediated via the paxillin pathway.^61^

**In conclusion,** our study shows that stretch and tachycardia induce early cardiac remodelling, including hypertrophy of myocytes and non‐myocytes. This is associated with mainly separate gene expression alterations and intracellular mechanisms in a cell type‐specific manner. Contribution of the NCX reverse mode is relevant to both triggers and might have clinical implications in stretch‐associated remodelling that occurs in cardiovascular diseases, such as in HFpEF or tachymyopathy.

### 4.6 Limitations

Pharmacological inhibitors always have the potential to effect more than one target and to lack specificity. In this report, we have proven this for KN93 which showed CaMKII independent effects by inhibiting a common off‐target protein together with KN92 in our settings. A candidate for this off‐target protein would be the IKr.^55^ For the NCX inhibitor KB‐R7943 which we used for a large portion of our results, we also cannot exclude potential unspecific effects, for instance on TRP channels^62^ Ca^2+^ channels^63^ and K^+^ channels.^64^ Novel NCX inhibitors are reported but were not available to us for performing experiments.

## CONFLICT OF INTEREST

We confirm that none of the authors of this manuscript has any conflicts of interest to disclose.

## AUTHOR CONTRIBUTION

ND, SLH, IM and KJ performed research. ND and EB analyzed the data and wrote the paper. EB designed the research study. DL, EK, AH, BL, SS, PR and SLH contributed financial resources, research reagents and tools and contributed to writing the paper.

## AUTHOR CONTRIBUTION

Natasa Djalinac: Conceptualization (equal); Formal analysis (lead); Investigation (lead); Methodology (equal); Validation (equal); Writing‐original draft (equal); Writing‐review & editing (equal). Senka Ljubojevic‐Holzer: Investigation (equal); Resources (equal); Writing‐review & editing (equal). Ingrid Matzer: Investigation (equal). Ewald Kolesnik: Resources (equal); Software (equal). Katharina Jandl: Investigation (equal). Birgit Lohberger: Resources (equal). Peter Rainer: Resources (equal). Akos Heinemann: Funding acquisition (supporting); Methodology (equal); Project administration (equal); Resources (equal); Supervision (equal). Simon Sedej: Resources (equal); Writing‐review & editing (equal). Dirk von Lewinski: Funding acquisition (lead); Project administration (equal); Resources (equal); Supervision (equal); Writing‐original draft (supporting); Writing‐review & editing (equal). Egbert Bisping: Conceptualization (lead); Formal analysis (equal); Methodology (equal); Project administration (equal); Resources (supporting); Supervision (equal); Writing‐original draft (lead); Writing‐review & editing (equal).

## Supporting information

Table S2

Table S3

Supplementary Material

## Data Availability

The data that support the findings of this study are available from the corresponding author upon reasonable request.
