# Supplementary material for: The role of stretch, tachycardia and sodium‐calcium exchanger in induction of early cardiac remodelling
Source: J Cell Mol Med. 2020 Jun 22;24(15):8732–43. doi: 10.1111/jcmm.15504 (PMC7412684; doi:10.1111/jcmm.15504)
Supplement: Supplementary file 3 — Supplementary Material [file JCMM-24-8732-s003.docx]

**Extended Material and Methods**

Neonatal rat cell culture

Neonatal rat ventricular cardiomyocyte culture (MC) was obtained from 1- to 3-days old rat hearts, which were digested at 37°C using 0.1% Liberase TL Research Grade (Sigma-Aldrich, now Merck) and 0.1% trypsin (Gibco - Thermo Fisher Scientific, Wilmington, USA). Pre-plating followed by BrdU treatment (100µM/L) was used to prevent proliferation of (potentially) remaining non-myocytes. To obtain a pure fraction of non-myocytes (NMC), the residual pre-plating cell fraction was collected and cultured in the absence of BrdU. Both MC and NMC were plated at a density of 2x10^6^ either on collagen type-IV pre-coated BioFlex cell culture plates (Dunn Labortechnik, Asbach, Germany) for cyclic stretch experiments or on 35 mm dishes (Grainer Bio-One, Kremsmuenster, Austria) coated with 1% gelatin for field stimulation. Cells were cultured in a serum-free medium for 3 days prior experiments/interventions. Cell contractility was preserved and regularly monitored using a conventional microscope during and after interventions.

Antagonist treatment

The MC were treated with 10µM FK-506 to inhibit Calcineurin, 10µM KN-93 to inhibit CaMKII, 10µM KB-R7934 to inhibit NCX, 10µM SKF-96365 to inhibit TRP channels, 10µM diltiazem to inhibit LTCC, 100µM strophanthidin to inhibit the Na^+^/K^+^ ATPase, 10µM losartan to inhibit the angiotensin II AT_1_ receptor, 10µM HOE-642 to inhibit NHE (Tocris, Bristol, UK) and 10µM flecainide to inhibit NaV 1.5 channels (Meda Pharma, Vienna, Austria). All chemicals were purchased from Sigma-Aldrich (now Merck), unless stated otherwise.

Quantitative Real Time PCR (qRT-PCR)

Cells were collected in a Qiazol^TM^ lysis reagent for RNA isolation. Total RNA purification was performed using the miRNeasy on a column isolation kit (Qiagen, Hilden, Germany) with DNase treatment. RNA yield was measured using the NanoDrop 2000 (Thermo Fisher Scientific, Wilmington, USA), while RNA quality was validated by the 2100 Bioanalyzer (Agilent Santa Clara, USA) and the QiaXpert (Qiagen, Hilden, Germany) setup. cDNA was synthesized using the QuantiTect Reverse transcription kit (Qiagen, Hilden, Germany) and used for qRT-PCR at the concentration of 10 ng per reaction volume.

MC Immunofluorescence Staining

MC cells were fixed in 4% PFA for 10 min at room temperature. Cells were permeabilized with 0.1% Triton-X-100 (Sigma-Aldrich, now Merck), washed with PBS, and incubated overnight at 4°Cwith primary antibodies: mouse monoclonal anti-desmin antibody (1:150, clone D33, DAKO, #M0760), rabbit polyclonal anti-CaMKII (phospho T286) antibody (1:200, Abcam, #ab32678), mouse monoclonal anti-Na^+^/Ca^2+-^exchanger antibody (1:200, SWANT, #R3F1), rabbit polyclonal anti-ADATS13 antibody (1:200, Abcam, cat. no. ab28274). Cells were washed twice with an antibody wash solution composed of PBS and 0.01% Triton-X-100, incubated with fluorophore-conjugated secondary antibodies for 2h at room temperature, and visualized on Zeiss LSM 510 Meta confocal microscope at 10x and 40x magnification.

NMC Immunofluorescence Staining

NMC fraction was detached from the cell culture flask using trypsin, and 0.2 x 10^5^ cells per well were seeded on an 8-well chamber slide. Cells were allowed to attach and were then fixed with ice-cold 1% PFA for 30 min. NMC cells were permeabilized with 0.2% saponin dissolved in PBS containing 3% BSA for 60 min at room temperature, and stained overnight at 4°C with the primary antibodies: goat polyclonal anti-DDR2 antibody (1:200, Santa Cruz, #sc-7555), mouse anti-vimentin antibody (1:1000, Abcam, #ab20346) and rabbit monoclonal anti - P4HB antibody (1:500, Abcam, #ab137110). Cells were incubated with fluorophore-conjugated secondary antibodies for 2 h and a mounting media with DAPI was added for the visualization on a Nikon A1 confocal microscope (Nikon, Japan). at 10x,20x,40x and 60x magnification.

Flow Cytometric Staining

NMC fraction was detached from the cell culture flask using trypsin and 0.5 x 10^6^ cells were evaluated by means of flow cytometric analysis. Cell viability was determined using eBioscience™ Fixable Viability Dye eFluor™ 506 (1:1000, Thermo Fisher Scientific, #65-0866-14) dissolved in PBS for 30 min at room temperature. Cells were then transferred into PBS, which contained 2% FBS and 1mM EDTA, and stained for 30 min at room temperature with the following antibodies: APC/Fire 750 anti-rat CD45 antibody (1:20, BioLegend #202221) and PE anti-rat CD31 antibody (1:50, Miltenyi Biotec, #130-116-505) Cells were washed two times, resuspended in PBS enriched with 2% FBS and 1mM EDTA.acquired on a Cytoflex S (Beckman Coulter).

Statistical analysis

Statistical significance between two groups was determined either by two-tailed *Student’s t-test* or by a non-parametric Mann-Whitney. One Sample T-test or Wilcoxon signed rank test was used when comparing qPCR fold-change to a control group with a pre-set value of 1. For treatments with a small sample size and unequal variance, an unpaired T-test with Welch corrections was used. When more than two groups were compared, two-way parametric and non-parametric ANOVA followed by the Bonferroni post-hoc was performed. The microarray dataset was statistically evaluated with two-way ANOVA followed by Benjamini-Hochberg false discovery rate correction. P-values of <0.05 were considered as statistically significant.

**Supplementary Table 1.** NRVCM qPCR oligonucleotide primer sequences.

| Target gene transcript | Oligonucleotide sequence | |
| --- | --- | --- |
| **ACTin, Alpha 1**  **(skeletal muscle) – ACTA1** | *Forward*  *Reverse* | 5‘-AGA GTC AGA GCA GCA GAA ACT AGA-3‘  5‘-CAC GAT GGA TGG GAA CAC AGC-3‘ |
| **Regulator of Calcineurin 1 –RCAN1** | *Forward*  *Reverse* | 5‘-GTG GCA AAC GGT GAT GTC TTC-3‘  5‘-CCC AGG AAC TCT GTC TTA TGC-3 |
| **Four and a Half LIM Domains 1 – FHL1** | *Forward*  *Reverse* | 5‘-TAC AGG CAG GGC TGG GTTTC-3‘  5‘-TGG AAA TGA GGT GTG GGC ATCT-3‘ |
| **Proatrial natriuretic peptide A - NppA** | *Forward*  *Reverse* | 5‘-ATC ACC AAG GGC TTC TTC CT-3‘  5‘-TGT TGG ACA CCG CAC TGT AT-3‘ |
| **Proatrial natriuretic peptide B - NppB** | *Forward*  *Reverse* | 5‘-AGT CGC TTG GGC TGT GAC GG-3‘  5‘-AAG AGC CGC AGG CAG AGT CA-3‘ |
| **GAPDH** | *Forward*  *Reverse* | 5‘-GCA ACT CCC ATT CTT CCA CCT TT-3‘  5‘-TAT CCT TGC TGG GCT GGG TG-3‘ |

**Supplemental Figures:**


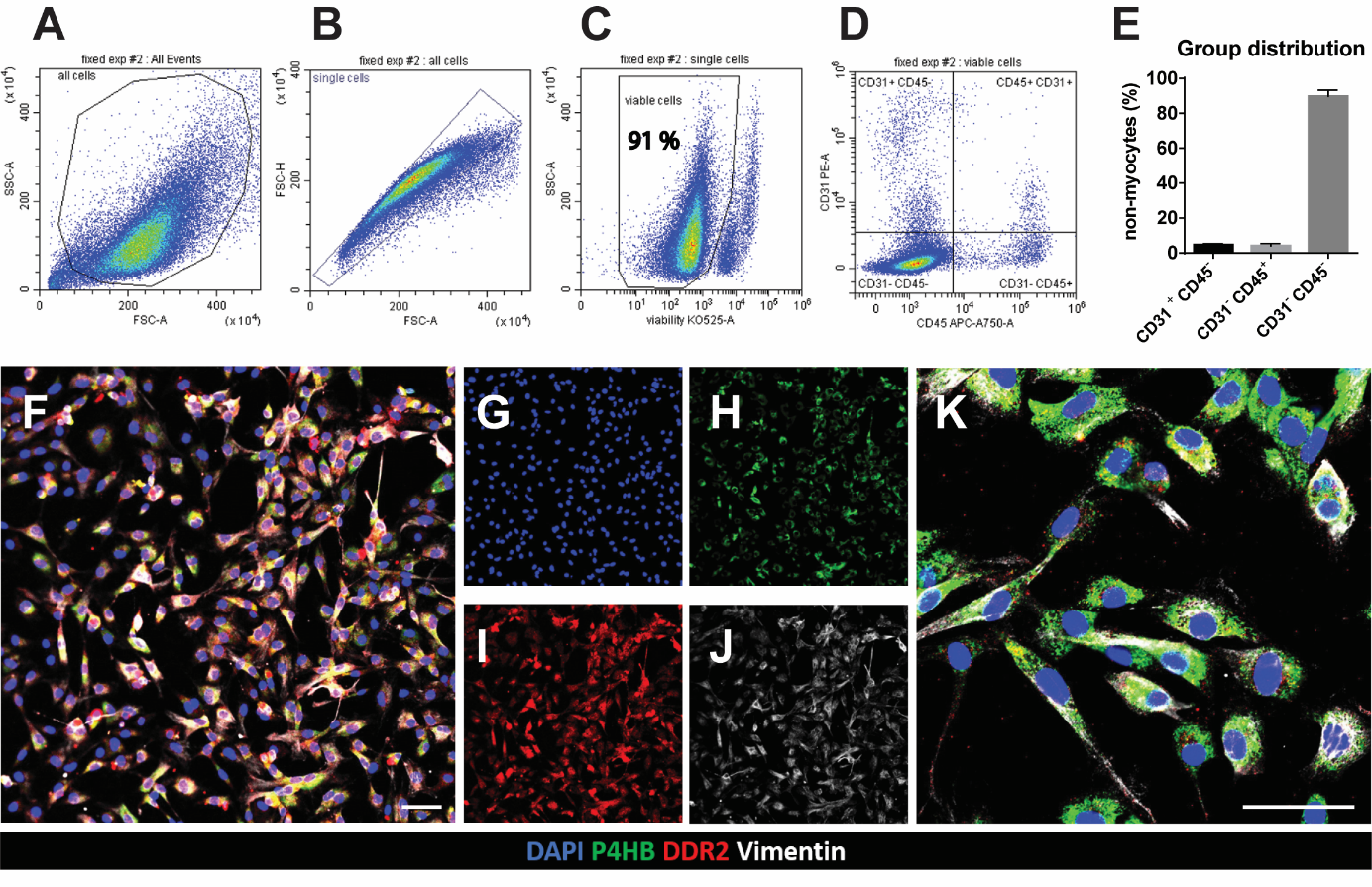


**Supplemental Fig. S1.** Cellular composition of non- myocytes (NMC). A-D. Representative Flow Cytometry plots showing gating strategy for (A) all events, (B) forward scatter for single cells, and (C) viable cells. (D) Cell clustering based on CD45 and CD31 surface markers. (E) Distribution of CD31^+^CD45^-^ , CD31^-^ CD45^+^ and CD31^-^CD45^-^ cells. (F) Immunofluorescent co-staining of NMC with (G) DAPI (nuclei) and P4HB, DDR2 and vimentin (fibroblast markers) (H-J). (K) Merged image of stained NMC. Scale bar = 50µm, n = 3 cell cultures.

To identify the specific composition of the NMC fraction, we first applied multicolor flow cytometry and identified a relative contribution of approximately 5% endothelial cells (CD31^+^CD45^-^) and 4% inflammatory cells (CD31^-^CD45^+^) of all viable cells (for gating strategy, please refer to Supplemental Fig.S1 (A-D), and for the quantification, please see Supplemental Fig.S1 (E). The majority of cells (90%) were negative for CD31 and CD45 suggesting mesenchymal fibroblasts. Since fibroblasts are heterogeneous cells, for which flow cytometric markers are as-of-yet not readily commercially available, we performed a triple color immunofluorescent staining of known fibroblast markers, namely P4HB, DDR2 and vimentin. All fibroblast markers stained the majority of cells, confirming our results obtained from the flow cytometric staining (Supplemental Fig.S1 F-K). The smallest CD31^+^CD45^+^ fraction likely represents progenitor cells.


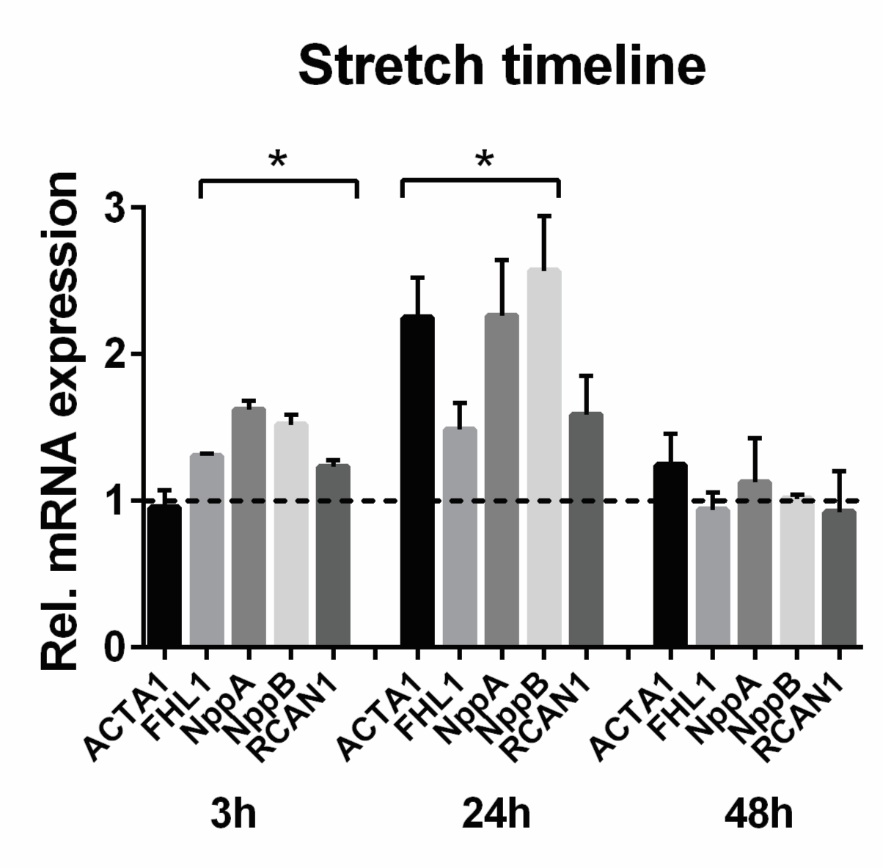


**Supplemental Fig. S2.** Time-dependent change of selected genes in response to stretch. Dashed line represents the baseline gene expression. ^*^p<0.05 *vs*. control, n=2 cell cultures (3 – 6 samples),





**Supplemental Fig. S3.** Time-dependent change of selected genes upon 24h of tachycardia (5Hz). Results are normalized to control group (1Hz). Dashed line represents the baseline gene expression. n=1 isolation (2 samples per group).


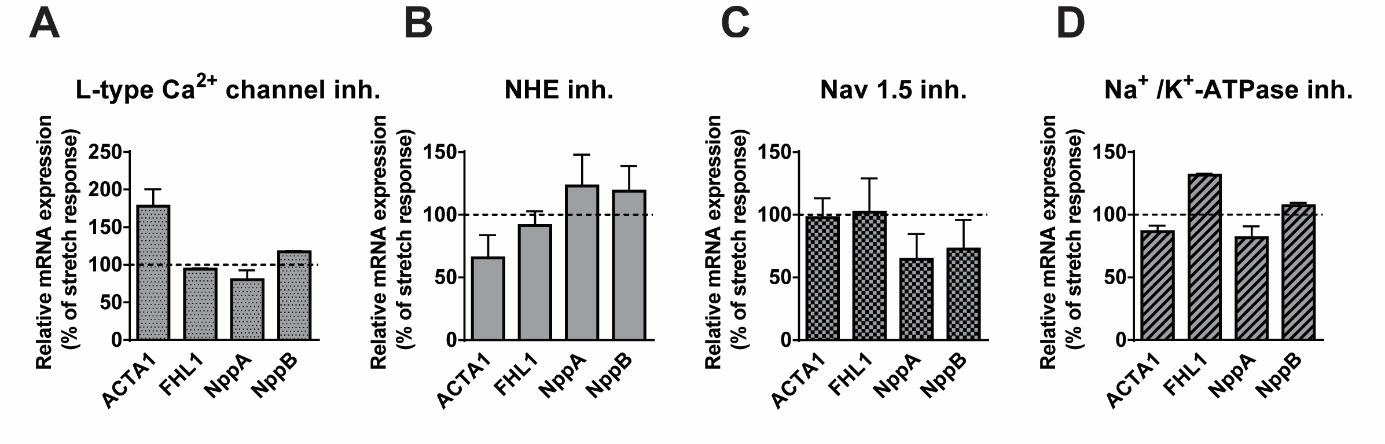


**Supplemental Fig. S4.** Gene expression after the inhibition of stretch (ST)-induced changes in MC. (A) diltiazem (L-type Ca^2+^ channel inhibitor), (B) HOE642 (the Na^+^/H^+^ exchanger inhibitor), (C) strophanthidine (Na^+^/K^+^ ATPase inhibitor) and (D) flecainide (NaV 1.5 channel blocker) did not alter the expression of pro-hypertrophy genes. Dashed line represents the maximum gene up-regulation under stretch conditions. Gene expression levels were normalized to respective controls. n=1-2 cell cultures.


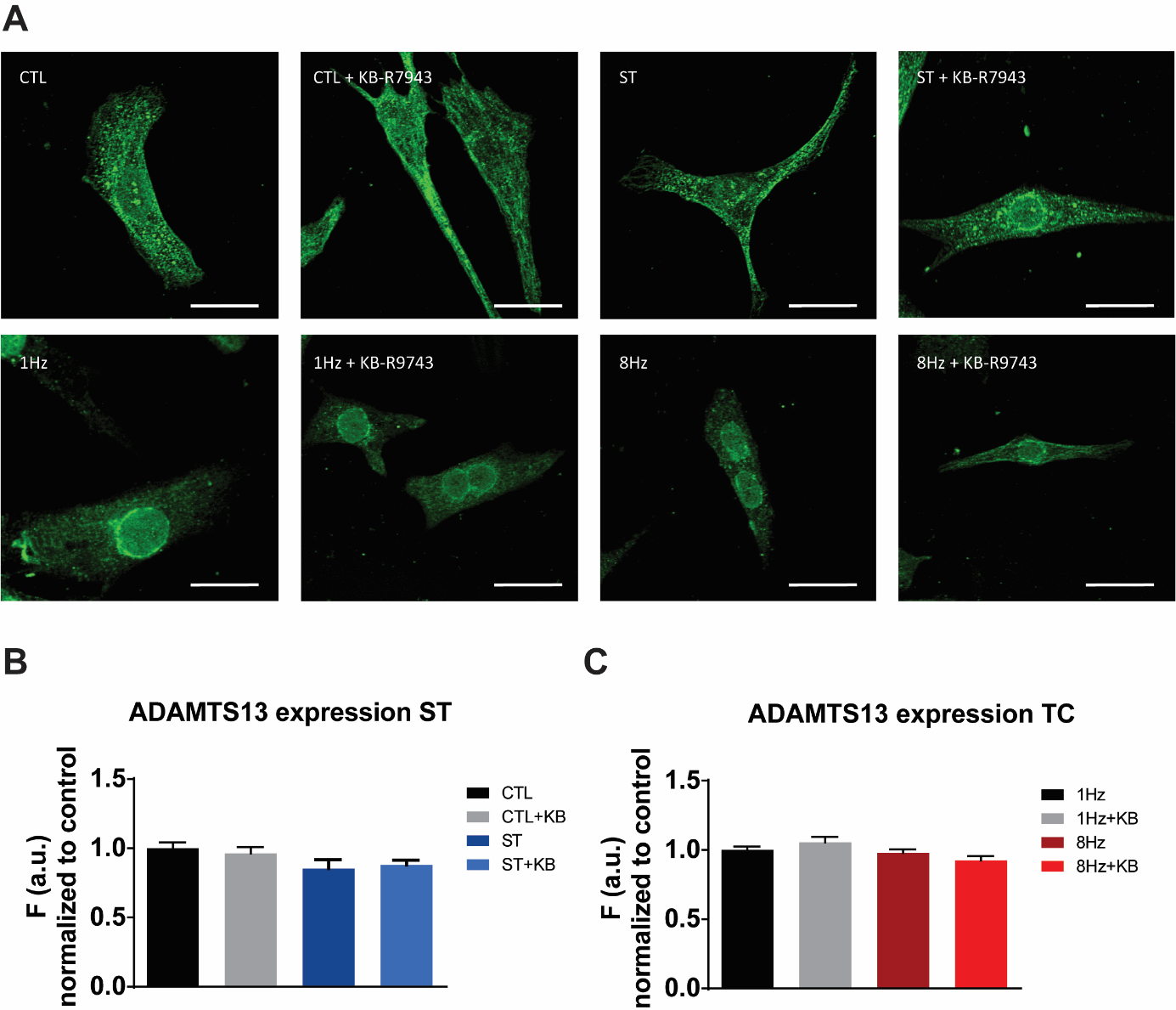


**Supplemental Fig. S5.** Expression levels of ADAMTS13 after 24h of stretch (ST) and 3h of tachycardia (TC). (A) Representative immunofluorescent staining of MC from bio-flex and 35mm dishes. Scale bar = 20 µm. (B-C) Protein levels after 24h of ST (B) or 3h of tachycardia (8Hz). (C) Average cell count n=1000 -1500 per group.
